# Study on the drunkenness of Chinese Baijiu with representative flavor based on behavioral characteristics

**DOI:** 10.3389/fnut.2022.1014813

**Published:** 2022-09-30

**Authors:** Xuefeng Guo, Yuxin Cheng, Yongguang Huang

**Affiliations:** ^1^College of Liquor and Food Engineering, Guizhou University, Guiyang, China; ^2^Key Laboratory of Fermentation Engineering and Biological Pharmacy of Guizhou Province, Guiyang, China

**Keywords:** Baijiu, representative flavor, behavioral characteristics, drunkenness degree, key drunkenness compounds

## Abstract

The essential role of drunkenness on the healthy development of Chinese Baijiu was studied in this research. This study revealed the effects of Baijiu on the behaviors of mice and evaluated the degree of drunkenness of soy sauce-, strong-, light-, and light and soy sauce-flavored Baijiu. The parameters obtained from the open field test were transformed into the behavioral drunkenness index by mathematical statistical analysis and the drunkenness-associated key compounds of Baijiu were analyzed. The results showed that strong- and light-flavored Baijiu presented higher levels of drunkenness and sobriety than soy sauce-flavored Baijiu. Interestingly, light and soy sauce-flavored Baijiu showed low drunkenness but a high sobriety degree. Specifically, the degree of drunkenness was positively correlated with fusel alcohol and aldehydes but negatively correlated with esters and acids. This study will enrich references for Baijiu behavior studies and lay a foundation for the research and development of healthy Baijiu.

## Graphical Abstract

There are key drunkenness compounds in Baijiu that can induce or reduce the degree of drunkenness, which lead to the differences in behavioral characteristics of mice after drinking Baijiu. 
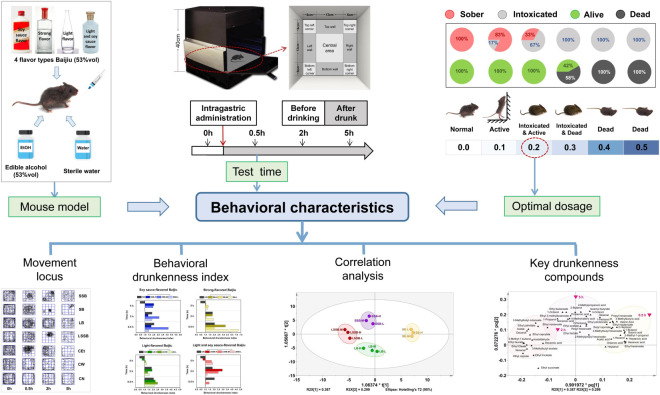

## Introduction

As a characteristic of traditional Chinese liquor, Baijiu has become an important product in daily life. Since 2016, the Baijiu industry has shown a trend of “volume reduction and efficiency increase” and has made a significant contribution to the economy (1). Based on the brewing process and flavor characteristics of Baijiu, 13 major flavors have been formed, such as traditional soy sauce-flavored Baijiu (SSB), strong-flavored Baijiu (SB), light-flavored Baijiu (LB), and innovative light and soy sauce-flavored Baijiu (LSSB) (2, 3). The characteristics of different flavors of Baijiu include the structure and concentration of compounds (4). In addition to flavor differences, the same flavor of Baijiu can also have significant differences in quality (5), which are closely related to the health attributes of Baijiu (6, 7). With the rational upgrading of consumption, the health attributes of Baijiu are of greater concern to consumers than its flavor characteristics. Thus, researchers gradually shifted their focus from flavor compounds to the degree of drunkenness and evaluation in Baijiu.

Previous studies have shown that animal models and behavioral tests are the classical methods used to study and evaluate the drunkenness of Baijiu. For example, Wu et al. established a linear regression model between comprehensive drunkenness and the behavior abilities of mice to evaluate the drunkenness of Baijiu (8). Xie et al. pointed out that acids, esters, fusel alcohol, and other principal components could regulate drunkenness by evaluating the drunkenness of different types of Baijiu in a mouse model (6). Wang et al. systematically evaluated the impacts of 20 non-alcoholic beverages on alcohol metabolism and the potential health benefits or harmful effects using mice as test models (9). Although these studies provided a theoretical reference for improving the health attributes of Baijiu, most of them focused on specific flavors of Baijiu or single behavioral parameters, lacking comprehensiveness. It was meaningful to focus on Baijiu with different flavors as well as the same flavor but different levels of quality as samples to comprehensively reveal the drunkenness degrees of Baijiu.

Our previous study found that the flavor characteristics and quality of Baijiu were systematically correlated with the kinds and contents of compounds by exploring the flavor structure of Baijiu (5). In this study, soy sauce-, strong-, light-, and light and soy sauce-flavored types of Baijiu were selected as the representative flavors of Chinese Baijiu. The method of animal behavior was used to study the degree of drunkenness of Baijiu, and the drunken mechanism was further explored by orthogonal partial least-squares discriminant analysis (OPLS-DA). This study revealed the internal mechanism of drunkenness differences, which could enrich references for Baijiu behavior studies and provide a theoretical basis for the health attributes of Chinese Baijiu.

## Materials and methods

### Baijiu samples

A total of 36 Baijiu samples produced in 2019 were purchased from 10 famous companies, including 9 samples of SSB (53% vol., produced by XJ, GT, and LJ); 9 samples of SB (52% vol., produced by LZ, WL, and YH); 9 samples of LB (53% vol., produced by FJ; 52% vol., produced by NL and BF); and 9 samples of LSSB (53% vol., produced by YB for 3 years). Based on the sensory flavor, characteristic volatile compounds structure, and price range of Baijiu (5), the above 36 Baijiu samples were divided into 3 categories: low quality (Low: market price from 100 to 400 RMB), middle quality (Middle: market price from 400 to 700 RMB), and high quality (High: market price from 700 to 1000 RMB).

Baijiu samples with the same flavor and quality were mixed in equal quantities (200 mL) to obtain 12 representative samples (4 different flavors, each flavor containing 3 levels of quality, Table 1).

**TABLE 1:** Baijiu samples used in animal experiment.

Number	Sample	Alcohol by volume
No. 1	Soy sauce-flavored Baijiu-low (SSB-L)	53.0 ± 1.0% vol.
No. 2	Soy sauce-flavored Baijiu-middle (SSB-M)	
No. 3	Soy sauce-flavored Baijiu-high (SSB-H)	
No. 4	Strong-flavored Baijiu-low (SB-L)	53.0 ± 1.0% vol.
No. 5	Strong-flavored Baijiu-middle (SB-M)	
No. 6	Strong-flavored Baijiu-high (SB-H)	
No. 7	Light-flavored Baijiu-low (LB-L)	53.0 ± 1.0% vol.
No. 8	Light-flavored Baijiu-middle (LB-M)	
No. 9	Light-flavored Baijiu-high (LB-H)	
No. 10	Light and soy sauce-flavored Baijiu-low (LSSB-L)	53.0 ± 1.0% vol.
No. 11	Light and soy sauce-flavored Baijiu-middle (LSSB-M)	
No. 12	Light and soy sauce-flavored Baijiu-high (LSSB-H)	

### Animal experiments

#### Animals

Male C57BL/6J mice (6–8 weeks, 18–22 g) were acquired from Wuhan Servicebio Technology Co., Ltd., China [Permission no. SYXK (Hubei) 2018-0101]. All mice were provided with a 12 h light/dark cycle (08:00–20:00) under specific pathogen-free conditions of controlled temperature (20–25°C), relative humidity (50–55%), and noise < 60 dB. Standard basic feed (SWS9102, Lab diet, Parker Migliorini International GmbH) and sterile water were used for adaptive feeding for a week. The body weight and food intake of the mice were recorded once a day at 09:00 a.m., and all mice were fasted for 12 h before gavage. All animal procedures were strictly performed according to the legislation for the care and use of laboratory animals of China and ratified by the Ethical Committee for Animal Experimentation of Guizhou University. The animal experiments were conducted in Human Provincial Laboratory Animal Center, Human Center for Safety Evaluation and Research of Drugs, Changsha, Hunan, People’s Republic of China.

#### Optimum gavage dosage of animal models

According to the safe alcohol dosage for adults prescribed by the World Health Organization (10) and the equivalent dosage of body surface area between humans (70 kg) and mice (20 g), the safe dosage of Baijiu (53% vol.) for mice was calculated to be 0.19 mL/d. A total of 70 mice were randomly assigned to 7 groups (*n* = 10 per group), including 3 control groups: CN (no treatment), CW (water), and CEt (53% edible alcohol), and 4 Baijiu groups: SSB-M (Soy sauce-flavored Baijiu-Low), SB-M (Strong –flavored Baijiu-Middle), LB-M (Light-flavored Baijiu-Middle), and LSSB-M (Light and soy sauce-flavored Baijiu-Middle). The CW, CEt, and Baijiu groups were given the same gradient dosages (0.1, 0.2, 0.3, 0.4, and 0.5 mL/20 g⋅bw).

The behavior and death situations of mice were observed by loss of righting reflex (LORR), and the rates of drunkenness and death within 5 h were calculated (8, 11). Drunken mice usually appear excited or depressed in mood, crawling unsteadily, dragging the floor with their hind legs, circling around, and exhibiting other abnormal behaviors (12, 13). The optimal dosage was selected based on climbing wall standing time (14, 15). An open field test system (25 × 25 × 40 cm, DigBehv) was used for the test before (0 h) or after gavage (0.5, 1.5, 2.5, 3.5, and 5 h). The mice were first placed in the open field for 1 min to acclimate, and then the climbing wall standing time within 5 min was recorded from the 2nd min. The optimal dosage and the time points at which the behavior of mice differed significantly were determined by LORR and climbing wall standing time.

#### Evaluation of the effects of Baijiu with different flavors on the behavior of mice

A total of 180 mice were randomly assigned to 15 groups (*n* = 12 per group), including 3 control groups: CN (no treatment), CW (water), and CEt (53% edible alcohol), and 12 Baijiu groups (SSB-L, SSB-M, SSB-H, SB-L, SB-M, SB-H, LB-L, LB-M, LB-H, LSSB-L, LSSB-M, and LSSB-H, see Table 1). The control and Baijiu groups were given the same dosage (0.2 mL/20 g⋅bw), which was selected based on the “Optimum gavage dosage of animal models” section above.

The mice were placed in the open field test system for 1 min to adapt to the environment before (0 h) or after gavage (0.5, 2, and 5 h), and the behavioral parameters within 5 min were recorded from the 2nd min. These parameters included total movement distance, central movement distance, central activity time, central stagnation time, and climbing wall standing time. After the behavioral test at each time point, the blood of 25% of the mice was immediately collected through eyeball extirpation. After the blood was incubated at 4 °C for 2 h and centrifuged at 3,000 r/min for 15 min, the upper serum was taken and stored at –80°C for further analysis.

### Evaluation of the drunkenness based on behavioral characteristics

The behavioral drunkenness index (BI) of mice was calculated according to the methods described in the literature and adjusted appropriately (6, 8, 11).


(1) $$\text{BI}=\frac{\text{CBts}}{\text{CBtn}}$$

CBts and CBtn represent the comprehensive behavioral capacity parameters of mice in the gavage groups (CW, CEt, and 12 Baijiu groups) and the no treatment group (CN), respectively.


(2) $$\text{CB}=\left(\text{EC}+\text{CC}\right)\times 50\%=\left[\text{EC}+\left(\frac{\text{RA}+\text{RE}}{2}\right)\right]\times 50\%$$

EC represents the exercise capacity of mice (the total movement distance ratio before and after gavage).

CC represents the cognitive capacity of mice (arithmetic mean of the anxiety parameter ratio (RA) and the exploration parameter ratio (RE) before and after gavage). Among them, the anxiety parameter is the central average velocity, and the exploration parameter is equal to the arithmetic mean of the central stagnation time and the climbing wall standing time.

### Statistical analysis

All data are presented as the mean ± standard error. Significant differences (*P* < 0.05) were evaluated with one-way analysis of variance and multiple comparisons of least-significant differences by SPSS Statistics 26. The classification prediction model was established by OPLS-DA using SIMCA 14.1. The variable importance for projection (VIP) was calculated according to the model, and the key variables were selected with VIP > 1 and *P* < 0.05 (16). Plotting analysis was conducted with Origin 8.5 and TBtools software.

## Results and discussion

### Gavage dosage of animal models

**FIGURE 1:**
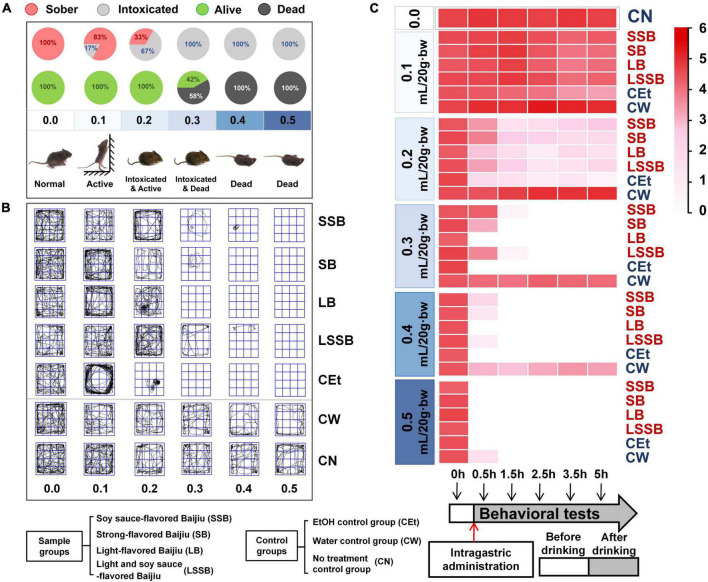
Effects of different gavage dosages on drunkenness degree of mice. **(A)** Drunken rate and death rate of mice; **(B)** movement locus; **(C)** climbing wall standing time in the open field. Experimental groups included: SSB, soy sauce-flavored Baijiu; SB, strong-flavored Baijiu; LB, light-flavored Baijiu; LSSB, light and soy sauce-flavored Baijiu; CEt, 53% edible alcohol; CW, water; CN, no treatment.

The rates of drunkenness and death (Figure 1A), movement locus (Figure 1B) and climbing wall standing time (Figure 1C) of mice at different time points were analyzed at the different dosages. Figure 1A shows that the mice were sober at the dose of 0 mL/20 g⋅bw, while the drunkenness rate was 17% at the 0.1 mL/20 g⋅bw dose and significantly increased to 67% at the 0.2 mL/20 g⋅bw dose. The drunkenness rate of mice at the 0.3 mL/20 g⋅bw dose reached 100%, and the death rate was 58%. At a dose of 0.4–0.5 mL/20 g⋅bw, all mice in the Baijiu and CEt groups died (this was considered a lethal dosage).

As shown in Figures 1B,C, the movement locus of sober mice (0.0 mL/20 g⋅bw) was evenly distributed, with a strong desire to escape, and the climbing wall standing time of sober mice was as high as 27.25 ± 0.68 s. Following gavage of 0.1 mL/20 g⋅bw, the movement locus in the surrounding area was increased, and the climbing wall standing time was prolonged (20.18 ± 1.25 s). At a dosage of 0.2 mL/20 g⋅bw, there was a significant difference in the movement locus between the different Baijiu groups (*P* < 0.05), and the climbing wall standing time of all mice decreased (22.20 ± 0.83 s to 8.20 ± 1.53 s). A total of 58% of the mice died at the 0.3 mL/20 g⋅bw dose, and the mice that did not die lost the capacity to climb the wall and stand after 1.5 h. Generally, the 0.2 mL/20 g⋅bw dose resulted in a high drunkenness rate (67%) without death, which could effectively reflect the drunkenness effect of mice with different flavors and quality levels of Baijiu. Thus, 0.2 mL/20 g⋅bw was determined to be the optimal dosage for subsequent experiments.

At a 0.2 mL/20 g⋅bw dose, the climbing wall standing time was used to select the time points with significant behavioral differences in mice (Figure 1C). The results showed that the average amount of climbing wall standing time decreased significantly at 0.5 h (from 22.20 ± 0.83 to 8.20 ± 1.53 s, *P* < 0.05, Figure 1C). The climbing wall standing capacity of the mice remained low from 1.5 to 5 h (2.68 ± 0.55 s). According to previous studies, the alcohol metabolism in mice was faster at 5 h than 2 h after drinking (8, 9). Thus, the representative time points were determined as before (0 h) and after gavage (0.5, 2, and 5 h) for the following animal experiments.

### Changes in the behavioral characteristics of mice after drunkenness

#### Effects of various Baijiu on the exercise capacity of mice

**FIGURE 2:**
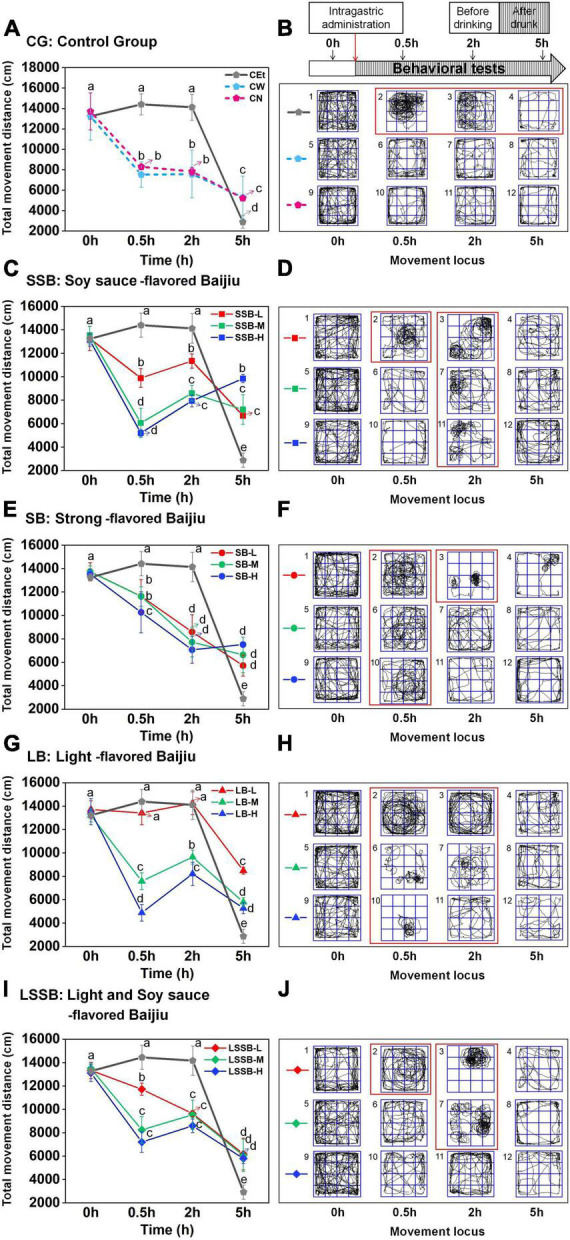
Effects of four representative flavors Baijiu intake on total movement distance and movement locus of mice. **(A,B)** Groups of CEt, CW, and CN; **(C,D)** group of SSB (soy sauce-flavored Baijiu); **(E,F)** group of SB (strong-flavored Baijiu); **(G,H)** group of LB (light-flavored Baijiu); **(I,J)** group of LSSB (light and soy sauce-flavored Baijiu). Experimental Baijiu can be divided into three levels of quality: low (L), middle (M), and high (H). Means with different letters were significantly different at *P* < 0.05. Error bars represent standard deviation (*n* = 3).

The total movement distance and locus of mice in the open field for 5 min were used as indicators to investigate the exercise capacity (13, 17), and the results are shown in Figure 2. At 0 h, all mice were excited (normal activity), with an average total movement distance of 13431.30 ± 847.30 cm (Figures 2A,C,E,G,I) and an evenly distributed movement locus (Figures 2B,D,F,H,J). At 0.5–2 h, the average total distance of each group was significantly different (*P* < 0.01, the lowest was 4881.86 ± 715.85 cm, the highest was 14407.39 ± 1025.18 cm), and the movement locus was chaotic and varied. Therefore, it could be indicated that the impacts of different flavors Baijiu on mouse behavior were mainly experienced at 0.5–2 h. The average total distance at 5 h was low (6302.64 ± 768.01 cm), but it showed the characteristics of uniform locus and wall-sticking activity, which was consistent with the observation of normal rats in the open field by Whishaw (18). It can be concluded that the drunken mice gradually recovered to the sober state at 5 h.

Both CEt and Baijiu inhibited the exercise capacity of mice, and Baijiu with different flavors or quality levels showed different levels of inhibition. Among the SSB groups, mice in the SSB-L group moved in circles at 0.5 h (Figure 2D2). Unlike the SSB-L group, the SSB-M and SSB-H groups showed no obvious circular clustering in the center until 2 h after gavage (Figures 2D3,7,11). Some similar studies have confirmed that alcohol intervention causes mice to exhibit abnormal movements, walking deficits, and decreased motor coordination (13, 17). These results concluded that the inhibitory effect of low-quality Baijiu on exercise capacity was stronger than that of high-quality Baijiu. All SB groups showed a large area of circular movement at 0.5 h (Figures 2F2,6,10), and the total movement distance (11175.29 ± 1900.42 cm) was significantly (*P* < 0.05) higher than those of the other Baijiu groups, explaining why SB had the strongest inhibitory effect on the exercise capacity of mice among the 4 flavors of Baijiu. In particular, the total distance of the LB-L group (13830.73 ± 631.434 cm) was close to that of the CEt group (14266.72 ± 1144.90 cm) at 0.5 h, which initially indicated that LB-L had a high similarity with CEt. Interestingly, the characteristics of LSSB were similar to those of SSB; LSSB-L showed a large circular movement at 0.5 h (Figure 2J2), while LSSB-M showed a significant circular movement at 2 h (Figures 2J3,7). There was no significance in the movement locus of LSSB-H during the whole process (Figures 2J9,12), indicating that LSSB-H had little influence on exercise ability.

Briefly, 4 representative flavors Baijiu had significant differences in the exercise capacity of mice from 0.5 to 2 h (*P* < 0.01), and the inhibitory effect of the SB group was stronger than those of the SSB, LB, and LSSB groups. In terms of the inhibitory effect on the exercise capacity of mice, low-quality Baijiu and CEt showed higher levels than high-quality Baijiu, which may be related to the key compounds that could reduce the effects of alcohol in the complex components of high-quality Baijiu.

#### Effects of various Baijiu on the cognitive capacity of mice

**FIGURE 3:**
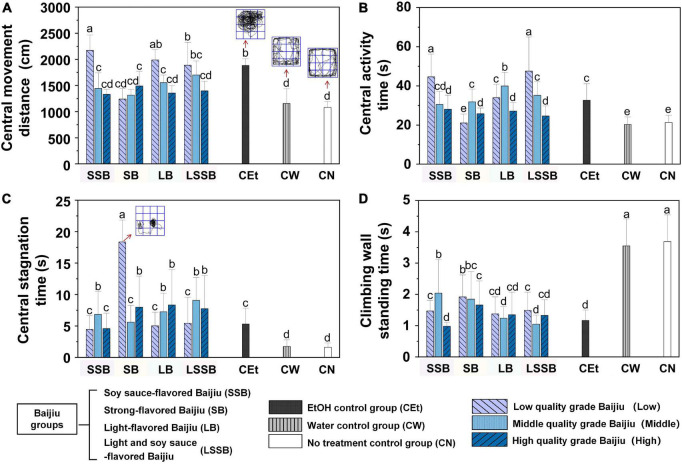
Effects of four representative flavors Baijiu intake on cognitive capacity of mice. **(A)** Central movement distance; **(B)** central activity time; **(C)** central stagnation time; **(D)** climbing wall standing time. Experimental groups included: SSB, soy sauce-flavored Baijiu; SB, strong-flavored Baijiu; LB, light-flavored Baijiu; LSSB, light and soy sauce-flavored Baijiu; CEt, 53% edible alcohol; CW, water; CN, no treatment. Experimental Baijiu can be divided into three levels of quality: low (L), middle (M), and high (H). Means with different letters were significantly different at *P* < 0.05. Error bars represent standard deviation (*n* = 3).

The parameters of anxiety behavior (central movement distance and activity time) and exploration behavior (central stagnation time and climbing wall standing time) of mice during the drunken period (0.5–5 h) are shown in Figure 3.

The results showed that the central movement distance and activity time of CEt (1883.36 ± 129.77 cm, 32.67 ± 8.44 s) were significantly higher than CW (1155.18 ± 282.56 cm, 20.27 ± 3.84 s) and CN (1080.22 ± 113.73 cm, 21.31 ± 3.69 s, Figures 3A,B), explaining why alcohol stimulation could increase anxiety levels. With regard to the anxiety level, the SB groups revealed no obvious anxiety reaction, and the SSB and LSSB groups showed a higher level than the LB groups. In the SSB, LB, and LSSB groups, the quality of Baijiu showed an opposite trend with the central movement distance and activity time, and the high-quality Baijiu groups were close to the CW and CN groups, suggesting that Baijiu quality was negatively correlated with anxiety level.

In particular, the SB groups in Figure 3C had obvious characteristics. The central stagnation time of the SB-L group (18.36 ± 3.47 s) was significantly (*P* < 0.01) increased, and the mice tended to move in circles in the central region (Figure 2F2). There was no significant difference among the SB-M, SB-H, and Baijiu groups with the same quality level. The results of LORR showed that the SB-L group showed abnormal phenomena, such as prolonged immobility and circling at approximately 0.5 h, which further explained that the drunkenness of the SB-L group had changed from anxiety to depression, revealing no obvious anxiety.

In terms of central stagnation time, all Baijiu groups showed a higher level than the CW (1.75 ± 1.06 s) and CN (1.62 ± 0.68 s) groups. This result suggested that Baijiu could weaken the exploration behavior of mice. With regard to the climbing wall standing time (Figure 3D), the CW (3.55 ± 1.86 s) and CN (3.69 ± 0.85 s) groups had significantly (*P* < 0.01) higher times than the CEt group (1.17 ± 0.33 s), while the Baijiu groups were between the CEt and normal groups (average value: 1.48 ± 0.57 s). Research has shown that exposure to external stimuli can increase anxiety-like behaviors. Stimuli have complex effects on open field behavior, with physical stimuli reducing activity and emotional stimuli increasing activity (19). These results strongly support the reasons for the significant differences in behaviors among the groups of Baijiu, CEt, CW, and CN in this study. It was further confirmed that Baijiu could inhibit the exploration capacity of mice, while edible alcohol had the strongest inhibitory effect of all treatments.

These results indicated that SSB, LB, and LSSB might positively promote the cognitive capacity (anxiety and exploration) of mice, while SB had the opposite inhibitory effect, which was in agreement with previous works on the cognitive capacity of mice (14, 20). In addition, this study found that the quality level of Baijiu was negatively correlated with the cognitive capacity of mice. This might be related to alcohol stimulating the body’s central nervous system, resulting in temporary disruptions of cognitive and physical control functions (12, 21). However, except for alcohol, Baijiu still contains some trace compounds produced in the process of fermentation, aging, and storage that can closely affect the impact of drunkenness (22, 23). Hence, there were compounds that superimposed or alleviated the drunkenness of different flavors of Baijiu and substances that stabilized mood or reduced alcohol stimulation in high-quality Baijiu.

#### Dynamic behavior changes of mice by the various Baijiu

**FIGURE 4:**
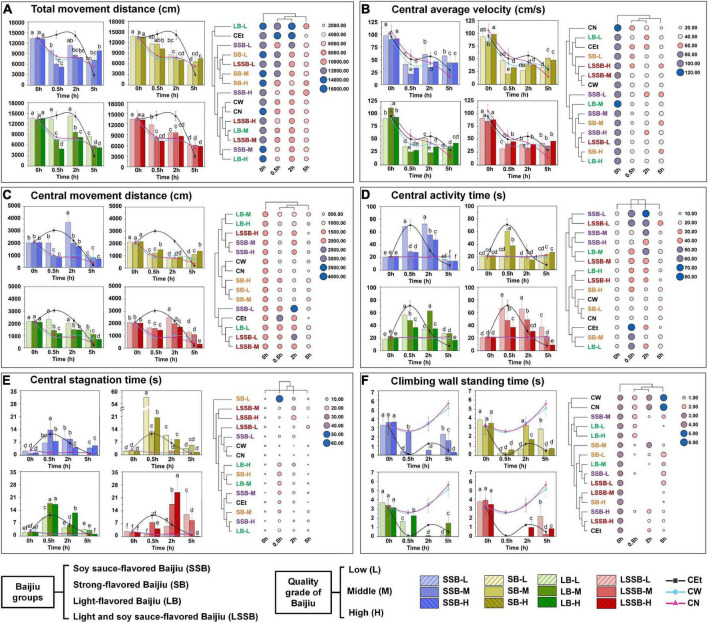
Dynamic changes on behavior of mice during the drunken period (0.5–5 h). **(A)** Total movement distance; **(B)** central average velocity; **(C)** central movement distance; **(D)** central activity time; **(E)** central stagnation time; **(F)** climbing wall standing time. Experimental Baijiu can be divided into three levels of quality: low (L), middle (M), and high (H). Means with different letters were significantly different at *P* < 0.05. Error bars represent standard deviation (*n* = 3).

To further reveal the difference in drunkenness time, this study tracked the dynamic changes in the main behavioral parameters of mice before (0 h) and after gavage (0.5, 2, and 5 h), as shown in Figure 4. The exercise capacity of mice was expressed as the change in total movement distance (Figure 4A). The middle-quality groups of SSB, LB, and LSSB were similar to the high-quality groups, and they were significantly (*P* < 0.01) different from the CEt, SB, and low-quality groups. The effect on exercise capacity of mice was mainly reflected after 0.5–2 h, with most of the mice returning to normal capacity at 5 h. In addition, the quality of Baijiu was negatively correlated with the injury degree of exercise ability. However, the SSB-H group was still drunk at 5 h, and the inhibition of exercise capacity of mice in the SSB-H group was higher than those in other groups, indicating that substances in SSB-H prolong the duration of drunkenness. As shown in Figure 4B, the central average velocity of all mice was high at 0 h (93.76 ± 12.84 cm/s) and gradually decreased to 36.65 ± 8.71 cm/s after adaptation, which was caused by the normal reaction of mice after entering the mode environment. Some similar phenomena have been confirmed by relevant studies (18).

The anxiety of mice was represented by the changes in central movement distance and central activity time (Figures 4C,D). The results indicated that the anxiety behavior of the SSB groups appeared at 2 h, and only the SSB-L group showed anxiety at 0.5 h. Although the anxiety behavior of the SB groups appeared at 0.5 h, the overall level was lower than those of the other Baijiu groups. The anxiety levels of the LB and LSSB groups were higher than those of the CN and CW groups from 0.5 to 2 h.

The exploration of mice was reflected by the central stagnation time (Figure 4E) and the climbing wall standing time (Figure 4F). The results showed that the central stagnation times of the SSB groups were lower than that of the CEt group, and their climbing wall standing capacity was strongly inhibited at 2 h. Compared with the times of the other groups, the central stagnation time of the SB group at 0.5 h was the highest, and the central stagnation time of the SB-L group was 5 times higher than that of the CEt group, while the climbing wall standing time was the shortest. The central stagnation times of the LB and LSSB groups were significantly (*P* < 0.05) increased at 0.5 h (14.99 ± 8.60 s) and 2 h (15.68 ± 8.87 s), and the climbing wall standing capacity decreased at 2 and 0.5 h, respectively. These results indicated that there were significant differences in drunkenness time among the 4 representative favors of Baijiu.

### Behavioral drunkenness index of mice treated with different types of Baijiu

**FIGURE 5:**
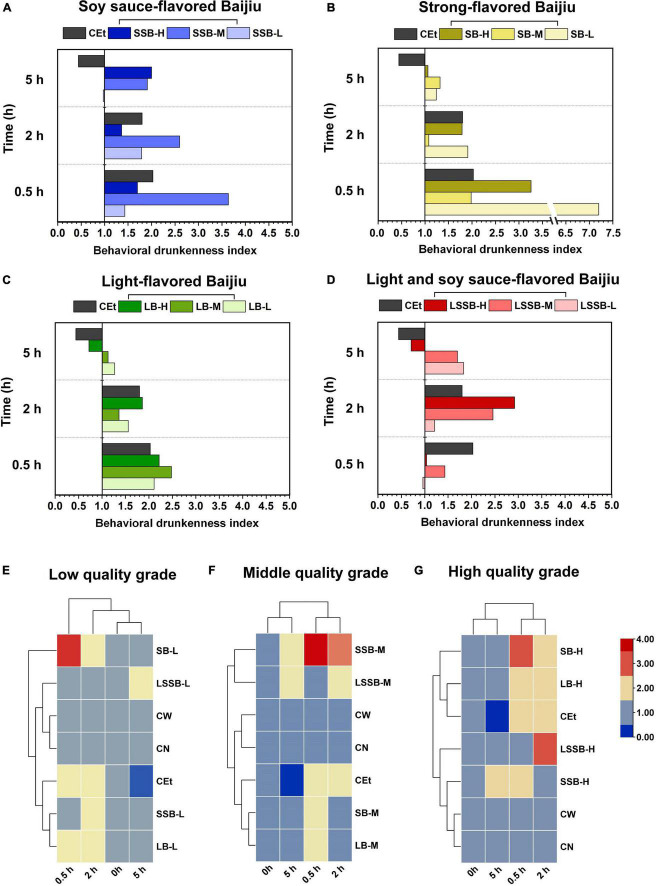
Behavioral drunkenness index analysis of mice during the drunken period (0.5–5 h). The behavioral drunkenness index groups included: **(A)** SSB, soy sauce-flavored Baijiu; **(B)** SB, strong-flavored Baijiu; **(C)** LB, light-flavored Baijiu; **(D)** LSSB, light soy sauce-flavored Baijiu; **(E)** L, low quality grade Baijiu; **(F)** M, middle quality grade Baijiu; **(G)** H, high quality grade Baijiu. The control groups included: CEt, 53% edible alcohol; CW, water; CN, no treatment.

The BI value was calculated according to the behavioral characteristics of the mice, and BI = 1.0 indicated a normal level in the CW and CN groups (Figure 5). The results showed that the BI values of the different Baijiu groups were significantly different after 0.5, 2, and 5 h (*P* < 0.05).

Among the SSB groups, the BI value of SSB-L showed a trend of increasing first and then decreasing with time, and the corresponding BI values were 1.43 ± 0.18 (0.5 h), 1.79 ± 0.19 (2 h), and 0.97 ± 0.02 (5 h), respectively (Figure 5A). Compared with the SB-L and LB-L groups, the SSB-L group had a lower BI value, which was due to the substances in the complex system of SSB that can reduce the drunkenness level. The BI value of the SSB-M group decreased from 3.64 ± 1.73 to 1.91 ± 0.14 from 0.5 to 5 h, which was higher than the values of other Baijiu groups of the same quality. With the increase in Baijiu quality, the types and contents of compounds in Baijiu increased, including the factors that superimposed drunkenness. These factors positively stimulated the behaviors of mice, resulting in a prolonged climbing wall standing time (Figure 4F). At 0.5–2 h, the BI value of the SSB-H group decreased from 1.70 ± 0.81 to 1.36 ± 0.44 and increased to 2.00 ± 0.11 after 5 h, which was higher than the times of other Baijiu groups with the same quality. Since most of the alcohol in mice has been metabolized (low BI value) by 5 h after drinking (8, 9), the BI value of the SSB-H group at 5 h was higher than those of the other groups in this study. It is reasonable to believe that the high content of macromolecular compounds metabolized by high-temperature fermentation in SSB (5) prolonged the metabolism of mice and caused slow sobriety.

The BI values of the SB groups showed a gradient downward trend over time (Figure 5B). At 0.5 h, the BI value of the SB-L group was higher than those of all of the other Baijiu groups and decreased from 7.22 ± 2.55 to 1.91 ± 0.42 after 2 h, which was close to that of the CEt group (1.80 ± 0.58). The results showed that in the SB-L group, some compounds superimposed with alcohol to aggravate and accelerate drunkenness. The SB-M group had a high BI value at 0.5 h (1.98 ± 1.01) that decreased to normal levels after 2 h (1.08 ± 0.63) and 5 h (1.32 ± 0.12), suggesting that the interactions of active compounds other than alcohol reduced the drunkenness effect (22, 23). Similarly, the SB-H group also had a high BI value at 0.5 h (3.26 ± 2.59) that gradually decreased to 1.06 ± 0.04 at 5 h. With the improvement in Baijiu quality, the compounds with mitigative effects increased, so the superposed effect of compounds and alcohol was weakened, and the SB-H group returned to normal after 5 h.

The variation tendency of the LB-L, LB-M, and LB-H groups was consistent with that of the CEt group; the BI value peaked at 0.5 h (2.11 ± 0.49, 2.48 ± 0.67, and 2.22 ± 0.67, respectively) and then gradually decreased (Figure 5C). It is known that the kinds and contents of compounds in LB are less than those in other flavors of Baijiu (5); therefore, the drunkenness is similar to that of simplex alcohol. At 5 h, the BI values of the LB-L, LB-M, and LB-H groups were close to those of the CW and CN groups, which were 1.27 ± 0.03, 1.13 ± 0.07, and 0.72 ± 0.03, respectively. At this time, alcohol metabolism in mice was almost finished, so the LB groups could sober up faster. Comparing Baijiu types with the same flavor, the compounds of high-quality Baijiu were more harmonious than those of low-quality Baijiu, and there were compounds that could reduce drunkenness.

The changes in the BI values in the LSSB groups are shown in Figure 5D. The BI value of the LSSB-L group gradually increased from 0.96 ± 0.12 (0.5 h) to 1.83 ± 0.12 (5 h), and that of the LSSB-M group increased from 1.43 ± 0.65 (0.5 h) to 2.46 ± 0.86 (2 h) and decreased gradually after 2 h. Similarly, the BI value of the LSSB-H group was normal at 0.5 h (1.04 ± 0.27) and increased to a peak at 2 h (2.92 ± 0.74), which was significantly higher than those of the other Baijiu groups (*P* < 0.05). The results showed that the drunkenness of LSSB was relatively slow (mainly occurring at 2 h). The BI values of the LSSB-L, LSSB-M, and LSSB-H groups (1.83 ± 0.12, 1.70 ± 0.19, and 0.71 ± 0.03, respectively) were lower than those of the other Baijiu groups at 5 h, which fully indicated that the LSSB groups showed slow drunkenness and rapid sobriety. Compared with the previous results, LB had the characteristics of fast digestibility with fewer drunken compounds, while SSB had more decelerating drunken compounds and slow drunkenness. Interestingly, LSSB had not only the characteristic of quick sobriety of LB but also the common characteristic of complex compounds of SSB, thus enabling the slow drunkenness and fast sobriety of mice.

The most obvious finding to emerge from the analysis was that the SSB groups during 2–5 h had low degrees of drunkenness and sobriety. The SB groups were drunk at 0.5 h and returned to normal at 2 h, indicating high degrees of drunkenness and sobriety. The duration of drunkenness in the LB groups was 0.5–2 h, with characteristics of high drunkenness and sobriety degree. The LSSB groups were drunk at 2 h and had gradually sobered up at 5 h, with significant characteristics of low drunkenness but a high degree of sobriety.

A strong relationship between drunkenness and metabolism has been reported in the literature, exhibiting differences in the regulation of anxiety and stress (24). This discrepancy could be attributed to the interactions of active components other than alcohol in Baijiu, and some compounds could induce or reduce drunkenness in the complex mixture system of Baijiu. Therefore, the basic types of drunkenness could be divided into Type I (alcohol-induced), Type II (compounds-induced), and Type III (compounds-reduced).

The results of clustering analysis showed that the BI values of the different quality Baijiu groups were similar at 0 and 5 h, indicating that the drunkenness of mice was relatively low at 5 h (Figures 5E–G). The low- and middle-quality types of Baijiu presented high levels of drunkenness at 0.5 h (Figures 5E,F), and the low-quality Baijiu was mainly characterized by type I alcohol-induced drunkenness due to the small number of compounds present in the Baijiu. However, the types of compounds in middle-quality Baijiu increased, and the factors that could induce or reduce drunkenness also increased, resulting in 2 types: I+II and I+III. The BI value of high-quality Baijiu showed a high level of drunkenness at 2 h (Figure 5G), indicating the slow drunkenness of high-quality Baijiu. As mentioned in the previous results, high-quality Baijiu contains high concentrations of complex compounds (5), and the compounds that induce or reduce drunkenness become complex accordingly; therefore, they were characterized by composite type I+II+III.

### Correlation analysis between the behavioral drunkenness index of mice and key compounds in different types of Baijiu

**FIGURE 6:**
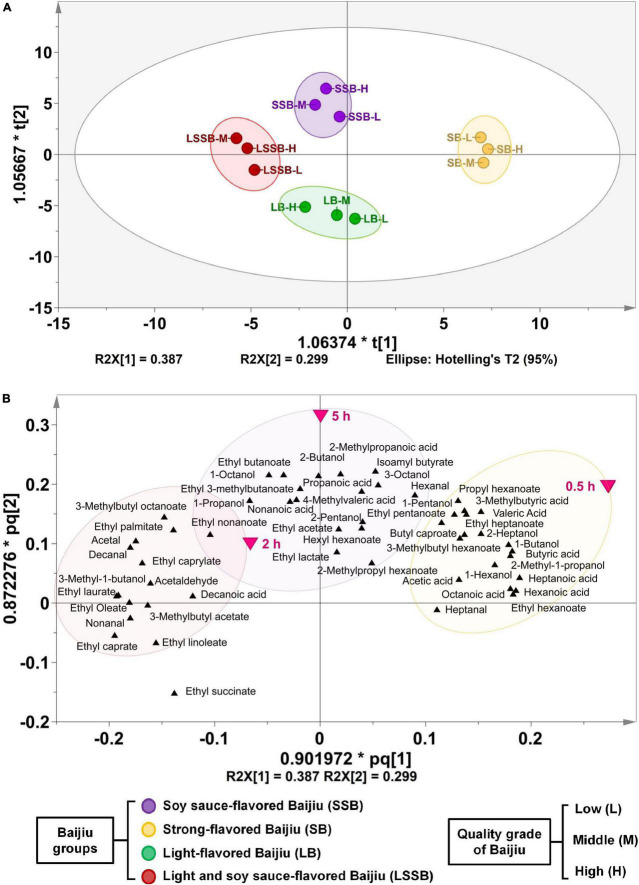
Correlation analysis of behavioral drunkenness index and key compounds in different Baijiu. **(A)** Score plot and **(B)** loading plot of partial least squares regression analysis. Experimental groups included: SSB, soy sauce-flavored Baijiu; SB, strong-flavored Baijiu; LB, light-flavored Baijiu; LSSB, light and soy sauce-flavored Baijiu; CEt, 53% edible alcohol; CW, water; CN, no treatment. Experimental Baijiu can be divided into three levels of quality: low (L), middle (M), and high (H).

Correlation analysis revealed that the drunkenness index of 4 representative Baijiu flavors had good correlations with key drunken compounds, among which SSB, SB, and LSSB were obviously divided into 3 categories. However, there were no compounds in LB that distinguished the degree of drunkenness (Figure 6).

The OPLS-DA model was used to further calculate the VIP value, and a total of 28 characteristic compounds were identified that contributed significantly to the distinction of drunkenness (Table 2). The results showed that the key compounds affecting the drunkenness degree at 0.5 h were mainly 5 esters, 5 acids, and 2 alcohols, which included ethyl hexanoate, butyric acid, and 1-butanol. The key compounds that played a role at 2 h were mainly 4 esters, including ethyl octanoate and ethyl laurate; 3 aldehydes, including acetaldehyde and acetal; and isoamyl alcohol and decanoic acid. Alcohol and most of the compounds in Baijiu were metabolized after 5 h, but there were still some positive factors that were difficult to metabolize or not completely metabolized, thus prolonging the duration of drunkenness. These factors included five esters and two acids, including ethyl lactate, ethyl acetate, and ethyl isovalerate, as well as nonanoic acid and 4-methylpentanoic acid.

**TABLE 2:** The variable importance for projection (VIP) values of PLS-DA model.

Compounds	VIP values	Compounds	VIP values
Isobutyl caproate	1.1877	Isoamyl alcohol	1.0871
Isoamyl caproate	1.1208	2-Methyl-1-propanol	1.0738
Butyl caproate	1.0917	1-Butanol	1.0292
Ethyl octanoate	1.0913	Nonanoic acid	1.0809
Ethyl laurate	1.0817	4-Methylvaleric acid	1.0791
Hexyl hexanoate	1.0665	Butyric acid	1.0543
Ethyl isovalerate	1.0636	Valeric acid	1.0344
Ethyl heptanoate	1.0590	Hexanoic acid	1.0225
Ethyl caprate	1.0526	Decanoic acid	1.0175
Ethyl acetate	1.0297	Octanoic acid	1.0125
n-Propyl hexanoate	1.0246	Heptanoic acid	1.0027
Ethyl hexanoate	1.0090	Decanal	1.0760
Ethyl nonanoate	1.0025	Acetaldehyde	1.0688
Ethyl lactate	1.0020	Acetal	1.0319

Based on the above characteristic compounds, we further analyzed the correlations between these compounds and the drunkenness degrees of the 4 representative Baijiu flavors. In this study, SB was positively correlated with “fusel alcohol” and other drunkenness factors. It is known that fusel alcohol (such as 2-methyl-1-propanol) in Baijiu might stimulate and anesthetize nerves, which could significantly increase drunkenness and even dizziness (6, 25). Therefore, SB showed a high degree of drunkenness at 0.5 h. In addition, the drunkenness of SB showed an opposite trend to the Baijiu quality, among which compounds such as ethyl caproate, hexanoic acid, and butyric acid increased with the Baijiu quality, thus playing a strong mitigative effect to reduce the drunkenness (7, 26).

This study found that fusel alcohol and aldehydes were positively correlated with SSB and LSSB, which may be related to the high levels of drunkenness of SSB and LSSB at 2 h. Clinical trials have proved that fusel alcohol and aldehydes have slow metabolism and long accumulation, which could cause a state of drunkenness characterized by blushing, dizziness, and unstable standing (9, 25, 27). Previous studies have noted the importance of certain compounds in Baijiu, such as esters, with the potential function of mood stabilization (28). Therefore, the levels of drunkenness of SSB and LSSB were slow due to the moderating effects of negative correlation factors such as ethyl octanoate and ethyl laurate.

In particular, the BI values were found to be high at 5 h in the SSB, LSSB-L, and LSSB-M groups. Since ethyl lactate might promote alcohol to stimulate the cerebral cortex and produce excitement (29), in this study, it was believed that alcohol and most of the compounds in Baijiu were metabolized by 5 h. However, there were still some positive factors that were difficult or incomplete to metabolize, such as ethyl acetate at high concentrations, resulting in a prolonged duration of drunkenness. Several reports have shown that ethyl acetate might eliminate harmful substances in Baijiu and reduce head pain after excessive intake (30). Meanwhile, suitable concentrations of organic acids can reduce head and taste discomfort and play a role in alleviating the degree of drunkenness (29, 31). Thus, the degrees of drunkenness of SSB and LSSB were lower than those of SB and LB, which may be closely related to the high contents of beneficial esters and acids that alleviated the drunkenness. Another important finding was that the LSSB-H group showed low drunkenness at 0.5 h and a high degree of sobriety at 5 h, which was speculated to be related to the characteristics of both SSB and LB. Meanwhile, we are conducting research on other possible characteristic compounds. The degrees of drunkenness with various Baijiu should also consider the effects of blood metabolism, intestinal microflora regulation, and body damage in further explorations to promote the healthy production of Baijiu.

## Conclusion

In summary, the degrees of drunkenness of representative Chinese Baijiu were analyzed based on the behavioral characteristics of mice at different times after intake. Both SB and LB presented higher levels of drunkenness and sobriety than SSB, while LSSB showed low drunkenness but a high sobriety degree. Meanwhile, the degree of drunkenness was negatively correlated with the quality of Baijiu. The correlations between drunkenness and key compounds showed that drunkenness was significantly affected by fusel alcohol, aldehydes, esters, and acids in Baijiu. Based on these effects, the types of drunkenness could be divided into Type I (alcohol-induced), Type II (compounds-induced), and Type III (compounds-reduced). This study provided new directions for the potential optimization and theoretical research of low-drunkenness Baijiu production.

## Data availability statement

The raw data supporting the conclusions of this article will be made available by the authors, without undue reservation.

## Ethics statement

This animal study was reviewed and approved by the Ethical Committee for Animal Experimentation of Guizhou University.

## Author contributions

YH and XG conceived the idea of the study. XG analyzed most of the data and wrote the initial draft of the manuscript. YH and YC contributed to refining the idea. All authors discussed the results and revised the manuscript.
